# Effects of aerobic exercise on TC, HDL-C, LDL-C and TG in patients with hyperlipidemia: A protocol of systematic review and meta-analysis

**DOI:** 10.1097/MD.0000000000025103

**Published:** 2021-03-12

**Authors:** Shaoping Zhao, Jiao Zhong, Caihong Sun, Junping Zhang

**Affiliations:** a Department of Teaching and Research of Physical Education, Wuhan Polytechnic University; b Department of Physical Education, Wuhan University of Technology; c Sports Department, Wuhan Institute of Technology; d School of Physical Education, Hubei University of Technology, Wuhan, China.

**Keywords:** aerobic exercise, hyperlipidemia, meta-analysis, protocol, systematic review, total cholesterol, triglyceride

## Abstract

**Background:**

With the development of the social level and the improvement of living standards, people's dietary structure changes in the direction of high blood fat, high sugar and high fever, which leads to the occurrence of many diseases.Long-term increase in blood lipids can easily cause cholesterol to invade the walls of large blood vessels, deposit and accumulate, and promote the proliferation of smooth muscle cells and fibroblasts in the arterial intima, leading to coronary heart disease and atherosclerosis (AS) and other cardiovascular and cerebrovascular diseases.

**Methods:**

Electronic databases including Google Scholar, PubMed, Web of Science(WOS), the Cochrane Library, EMBASE and VIP Database for Chinese Technical Periodicals, China National Knowledge Infrastructure, and Wanfang. These databases will be searched to identify randomized controlled trials published January 1, 1980, and January 20, 2021. Language is limited with English and Chinese. We will use the standards provided in Cochrane Handbook 5.3.0 for quality assessment and risk assessment, and use Revman 5.3 software for meta-analysis. The primary outcomes are mainly evaluated by total cholesterol and triglyceride.

**Conclusion:**

The results of this study can provide a beneficial basis for the improvement of total cholesterol, high density lipoprotein cholesterol, low density lipoprotein cholesterol, and triglyceride in patients with hyperlipidemia.

## 1 Introduction

With the development of the social level and the improvement of living standards, people's dietary structure changes in the direction of high blood fat, high sugar and high fever, which leads to the occurrence of many diseases. Among them, hyperlipidemia refers to a situation where the mass metabolism disorder caused by various reasons increases blood cholesterol or triglyceride levels. Long-term increase in blood lipids can easily cause cholesterol to invade the walls of large blood vessels, deposit and accumulate, and promote the proliferation of smooth muscle cells and fibroblasts in the arterial intima, leading to coronary heart disease and atherosclerosis and other cardiovascular and cerebrovascular diseases. Recent evidence from epidemiological investigations and animal experiments shows that hyperlipidemia may damage the blood-brain barrier, leading to severe damage to brain structure and function, and decreased hippocampal-dependent learning and memory capabilities.^[1]^ Although drugs can treat hyperlipidemia, long-term medication will not only cause side effects, but also increase the economic burden.

Studies have shown that aerobic exercise can affect the metabolism of blood lipids, as well as affect various indicators related to lipid metabolism. Exercise improves serum lipids in patients with hyperlipidemia by lowering serum triglyceride levels, total cholesterol (TC), and low-density lipoprotein cholesterol (LDL-C) levels, while increasing high density lipoprotein cholesterol (HDL-C) levels.^[2–4]^ Prior research indicates that lipoprotein lipase is critical in the formation of HDL, and is increased with aerobic exercise.^[5,6]^ Even intermittent or several short exercise sessions can positivel alter serum lipids. In sedentary individuals, lipoprotein and lipid changes can even occur after a single exercise session when one expends at least 350 kcal.^[7,8]^ Both cross-sectional and training intervention studies, suggest a beneficial impact of physical activity on both body composition, and lipid profile (particularly HDL-cholesterol and triglycerides) among elderly people.^[9–14]^ Therefore, aerobic exercise may have a positive impact on the blood lipid level of patients with hyperlipidemia, and may have a high effect as an adjuvant treatment.

## 2 Study aim/objective

The aim of our study is to propose a protocol for a systematic review and meta-analysis to assess the effectiveness of aerobic exercise on TC, HDL-C, LDL-C, and triglyceride (TG) in patients with hyperlipidemia.

## 3 Methods

This protocol has been checked with preferred reporting items for systematic review and meta-analysis protocols checklist.^[15]^ Meanwhile, Cochrane Handbook for Systematic Reviews of Interventions will be used as guidance to conduct this systematic review.^[16]^ Our protocol has been registered on the International Platform of Registered Systematic Review and Meta-Analysis Protocols (INPLASY). The registration number was INPLASY202120037 (10.37766/inplasy2021.2.0037).

### 3.1 Inclusion criteria

#### 3.1.1 Type of studies

Only randomized controlled trials(RCT) evaluating the effectiveness of aerobic exercise on cholesterol and triglycerides in patients with hyperlipidemia were included in this review.

#### 3.1.2 Participants

Serum TC ≥ 5.20 mmol/L, TG ≥ 1.7 mmol/L, high-density lipoprotein cholesterol (HDL-C) ≤1.04 mmol/L, LDL-C ≥ 3.61 mmol/L, no major disease, no blood lipid-lowering drugs in the past six months. All patients in the experimental group signed an informed consent form voluntarily to participate and were able to exercise as required.

#### 3.1.3 Intervention

In the experimental group, all patients must perform aerobic exercises, including walking, cycling, strength training, taiji, yoga, etc. to improve TC, HDL-C, LDL-C and TG levels in patients with hyperlipidemia.

#### 3.1.4 Comparator

The control group did not implement intervention, and did not receive other hypolipidemic treatments.

### 3.2 Information sources

Electronic databases including Google Scholar, PubMed, Web of Science(WOS), the Cochrane Library, EMBASE and VIP Database for Chinese Technical Periodicals, China National Knowledge Infrastructure, and Wanfang. These databases will be searched to identify RCTs published January 1, 1980, and January 20, 2021. Language is limited with English and Chinese.

### 3.3 Search strategy

The plan searched terms are as follows: “high blood lipid” or “hyperlipidemia” or “cholesterol” or “triglyceride” or “HDL-C” or “LDL-C” and “aerobic exercises” or “aerobic fitness” or “walking” or “cycling” or “strength training” or “taiji” or “yoga.”

### 3.4 Types of outcomes

#### 3.4.1 Primary outcomes

The primary outcomes are mainly evaluated by TC and triglycerides.

#### 3.4.2 Secondary outcome

The secondary outcomes are assessed by HDL-C, LDL-Cl, ApoA1, and ApoB indexes.

### 3.5 Data collection and analysis

#### 3.5.1 Study selection

**Figure 1:**
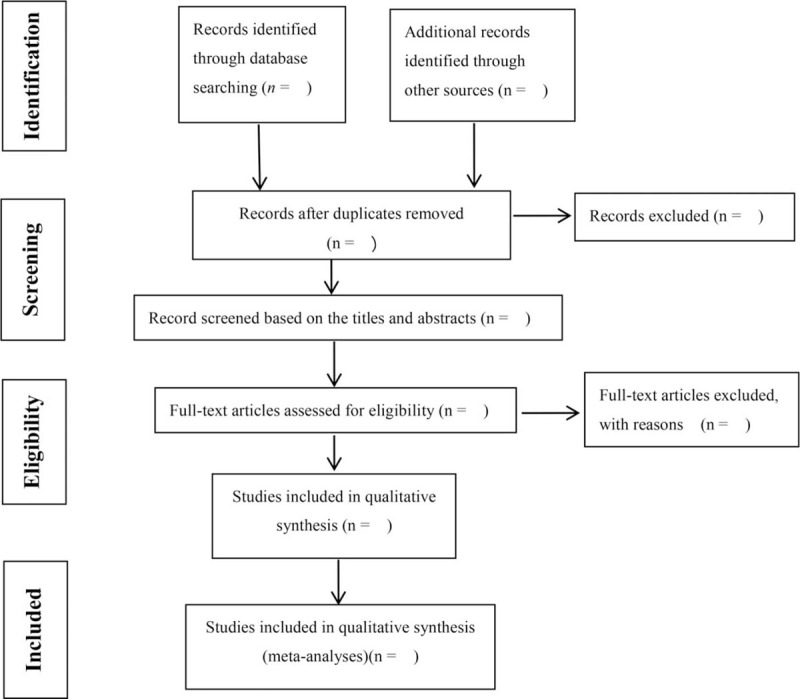
Study selection flow diagram.

The 2 authors (Zhao SP and Zhong J) will be independently reviewed to determine potential trials by evaluating the title and abstract. The full text will be further reviewed to exclude irrelevant studies or to identify potentially qualified studies. The disagreement between the two authors will be resolved through discussions with the third researcher (Sun CH). Endnote X7 software will be used for document management and record retrieval. The preferred reporting items for systematic reviews and meta-analysis conform to the flowchart (Fig. 1) will be used to describe the selection process of qualified literature.

#### 3.5.2 Data extraction

According to the data extraction table in Cochrane Reviewer's Handbook 5.3.0, two researchers will extract the following detailed information:

(1) Study characteristics:title, authors name, journal, publication year, country and follow-up duration, etc.
(2) Participants: gender, age, duration of disease, pain spots.
(3) Interventions: exercise type, intensity, exercise frequency, exercise time of every time, total exercise time.

#### 3.5.3 Dealing with missing data

When the data is lost or insufficient, we will contact the corresponding author via email to obtain the data. If these relevant data are not available, we will only analyze the available data and discuss its impact and limitations.

### 3.6 Quality assessment

Two experienced authors (Zhao SP and Zhong J) will evaluate the methodological quality of randomized controlled trials according to the RCT quality evaluation criteria recommended in Cochrane Reviewer's Handbook 5.3.0. This assessment will consist of 9 items: generation of the random sequence, allocation concealment, blinding of participants and investigator, similar baseline, = <15% dropouts, intention-to-treat analysis, between-group comparison, point measure and measures of variability. The higher the score, the better the quality of the method. Any disagreements will be resolved via discussion with a third researcher (Zhang JP).

### 3.7 Data synthesis and analysis

We will use Revman 5.3 software for meta analysis. The relative risk (RR) with 95% confidence interval is used for dichotomous variables, and the weighted average difference or standard mean deviation with 95% confidence interval is used for continuous variables.

### 3.8 Subgroup and meta-regression analysis

We will conduct subgroup and meta-regression analysis to explore the sources of heterogeneity such as age, gender, disease course, intervention type, research quality, exercise intensity, frequency of use, and exercise time.

### 3.9 Sensitivity analysis

Sensitivity analysis will be performed to assess the reliability and robustness of the results. By changing the inclusion criteria, excluding low-quality studies, using different statistical analysis methods to observe changes in RR. If the document that has a significant impact on the combined RR is excluded, the document is considered to be sensitive to the combined RR, otherwise it is not.

### 3.10 Publication bias

If ten or more studies are included in the meta-analysis, a funnel chart will be drawn and generated to assess potential publication. The asymmetry of the funnel chart shows publication bias. When necessary, we will use the Beggar test.

### 3.11 Evidence evaluation

The evidence grade will be determined according to the Grading of Recommendations, Assessment, Development, and Evaluation. The quality of evidence will be divided into high, medium, low, and very low.

## 4 Discussion

In recent years, the intervention form of aerobic exercise has been widely used in various diseases. This article reviews the effects of aerobic exercise on the blood lipids of patients with hyperlipidemia, exploring the positive effects of increased HDL cholesterol and training on hypertriglyceridemia. Aerobic exercise may have a beneficial effect on the composition of LDL. Some studies have shown that small density LDL particles and Apo B100 decrease.^[17–19]^ Similarly, Hypertriglyceride is an indicator of abnormal blood lipids and blood glucose, and has been proved to be an independent risk factor.^[20]^ Recently, a large number of studies have explored the effect of aerobic exercise on TC and triglyceride in patients with hyperlipidemia. Due to the different sample, time, intensity, frequency, exercise form and duration of randomized controlled trials, the specific effects of aerobic exercise on blood lipid level of patients with hyperlipidemia may be different. Therefore, the purpose of our meta-analysis was to evaluate the specific effects of aerobic exercise on TC and triglycerides in patients with hyperlipidemia. At the same time, this study will also explore the internal regulatory mechanism of aerobic exercise on hyperlipidemia, and provide corresponding exercise prescription for patients with hyperlipidemia.

## Author contributions

Shaoping Zhao and Jiao Zhong contributed to the conception; Caihong Sun and Junping Zhang applied the search strategy and performed the data analysis; Shaoping Zhao wrote this manuscript. All authors have read and agreed to publish the manuscript.

**Conceptualization:** Shaoping Zhao, Junping Zhang.

**Data curation:** Shaoping Zhao, Jiao Zhong, Junping Zhang.

**Formal analysis:** Jiao Zhong, Junping Zhang.

**Methodology:** Shaoping Zhao, Caihong Sun.

**Project administration:** Shaoping Zhao.

**Resources:** Caihong Sun.

**Software:** Shaoping Zhao.

**Supervision:** Caihong Sun.

**Validation:** Jiao Zhong.

**Writing – original draft:** Shaoping Zhao.

**Writing – review & editing:** Shaoping Zhao, Caihong Sun, Junping Zhang.
